# The biological memory buffer hypothesis: can programmable nanocarriers shape cellular memory to improve the durability of mRNA vaccines and gene therapies?

**DOI:** 10.3389/fddev.2026.1902184

**Published:** 2026-07-24

**Authors:** Okechukwu Paul-Chima Ugwu, Chinyere Nneoma Ugwu, Godson Emeka Anyanwu, Mariam Basajja, Maryann Chiamaka Ebuoh

**Affiliations:** 1 Department of Research, Publication and Extension, Kampala International University, Kampala, Uganda; 2 Department of Anatomy, Faculty of Biomedical Sciences, Kampala International University, Kampala, Uganda; 3 Department of Human Anatomy, State University of Medical and Applied Sciences, Igbo Eno, Enugu State, Nigeria

**Keywords:** biological memory, gene therapy, immune memory, lipid nanoparticles, mRNA vaccines, nanomedicine, RNA therapeutics, therapeutic durability

## Abstract

**Introduction:**

Messenger RNA vaccines and gene therapies enable rapid and programmable biological intervention, but their therapeutic durability remains variable. Existing nanomedicine research primarily evaluates delivery efficiency, targeting, RNA protection and early protein expression, which do not fully explain why some biological effects persist while others rapidly decline.

**Methods:**

This Hypothesis and Theory article presents a narrative conceptual synthesis of peer-reviewed literature on lipid nanoparticle delivery, mRNA vaccinology, RNA therapeutics, genome editing, trained innate immunity, epigenetic regulation and regenerative medicine. Evidence was integrated to develop an operational and falsifiable framework for evaluating the contribution of nanocarriers to therapeutic durability.

**Results:**

We propose the Biological Memory Buffer Hypothesis, according to which programmable nanocarriers may influence biological systems that encode, maintain or recall therapeutic information after RNA delivery. The framework distinguishes payload persistence from biological-outcome persistence and introduces three measurable constructs: therapeutic memory engineering, nanocarrier memory capacity and the therapeutic persistence window. It further proposes matched-cargo and matched-early-exposure experiments, candidate monophasic and biphasic decay models, a minimum durability reporting set and safety monitoring for maladaptive innate immune imprinting.

**Discussion:**

The framework does not assume that adaptive immune memory, trained immunity, epigenetic regulation and regenerative repair share a single molecular mechanism. Rather, it treats them as distinct biological substrates with a common functional relevance to durability. The hypothesis is falsifiable: failure to detect reproducible carrier-attributable differences under matched conditions would argue against nanocarrier memory capacity as an independent determinant. Incorporating longitudinal, tissue-specific and host-stratified durability measurements may support the rational development of longer-lasting RNA vaccines and gene therapies.

## Introduction

The clinical success of mRNA vaccines has made RNA nanomedicine one of the clearest examples of how drug delivery science can reshape medicine. Lipid nanoparticle platforms helped overcome long-standing barriers in nucleic acid therapeutics, including enzymatic degradation, poor cellular uptake and limited cytosolic access. In practical terms, they moved mRNA from a fragile experimental molecule into a deployable therapeutic class (Cullis and Felgner, 2024; Hou et al., 2021; Pardi et al., 2018). The field has since moved beyond infectious disease vaccination into oncology, protein replacement, immune modulation, genome editing and regenerative medicine (Huang et al., 2022; Rohner et al., 2022; Wu et al., 2024). That said, a delivery event is not the same thing as a durable therapeutic effect.

The problem is easy to see in vaccine studies. Antibody titres may fall even while memory B cells and T cells remain detectable, and boosters can improve recall without necessarily giving permanent circulating protection (Arunachalam et al., 2023; Goel et al., 2021; Turner et al., 2021). A similar issue appears in RNA-guided gene therapy. The delivery event may be transient by design, especially when the aim is to limit editing-enzyme exposure, but the clinical goal still depends on sustained correction, durable protein restoration or stable cellular reprogramming (Laurent et al., 2024; Musunuru et al., 2025). The practical question is therefore no longer just whether RNA can be delivered. It is whether the biological system can retain, stabilise and recall the therapeutic instruction.

It is useful at the outset to separate two senses of persistence that are easily conflated. Payload persistence refers to how long the delivered RNA, and the protein or editing activity it directs, remains present. Biological-outcome persistence refers to how long the therapeutic effect endures, whether through circulating protection, the survival of corrected cells or a stabilised tissue state. These can diverge sharply. *In vivo* base editing, for example, is deliberately transient at the payload level yet aims for permanent outcome persistence through a durable genomic change. Throughout this article, durability refers to outcome persistence unless payload persistence is specified.

Most nanomedicine frameworks still describe nanoparticles mainly as vehicles. Their performance is usually judged through particle size, polydispersity, encapsulation efficiency, surface charge, biodistribution, uptake, endosomal escape and early protein expression. These measures are necessary. They are also incomplete. Cells do not simply receive RNA and make protein in a neutral background. They interpret RNA delivery through innate immune sensing, antigen presentation, metabolic tone, chromatin state, tissue inflammation and repair history (Alameh et al., 2021; Lee et al., 2023; Sharma et al., 2024; van Leent et al., 2021). This interpretive layer is where the next conceptual advance in RNA nanomedicine may come from.

This article introduces the Biological Memory Buffer Hypothesis. The hypothesis proposes that programmable nanocarriers can shape the persistence of RNA therapeutic effects by interacting with biological memory systems. These systems include adaptive immune memory, trained innate immunity, epigenetic regulation, tissue-resident cellular states, stem-cell niche behaviour and regenerative repair programmes. The argument is not that a nanoparticle can simply switch memory on or off. Biology is much messier than that. Rather, therapeutic durability should be treated as an active design problem, not as a secondary observation made after delivery has already been optimised.

### Sources and approach

This article is a narrative conceptual analysis. It draws on peer-reviewed literature on lipid nanoparticle delivery, mRNA vaccine immunology, RNA therapeutics, CRISPR-based gene therapy, trained immunity, epigenetic regulation and nanomedicine-enabled immune modulation. Preference was given to primary studies in RNA, lipid nanoparticle and immunology research, together with authoritative reviews, indexed by major databases or hosted by established publishers (Cullis and Felgner, 2024; Hofstraat et al., 2025; Kulkarni et al., 2021; Wu et al., 2024). Older foundational studies were retained only where they remain central to RNA therapeutic development. The purpose is not to present the Biological Memory Buffer Hypothesis as an established clinical mechanism. Instead, the goal is to offer a testable framework for interpreting durability in RNA vaccines and gene therapies. A strong peak response after mRNA delivery may be useful, but it should not be mistaken for memory. Memory requires evidence of persistence, recall, functional stability or long-term biological reprogramming.

### Bounding the memory category

Before developing the hypothesis, we address a legitimate objection: adaptive immune memory, trained innate immunity, epigenetic state and regenerative repair operate on different cellular substrates, timescales and measurement modalities, so treating them as one phenomenon risks a category error. We accept this point and bound the framework accordingly. The “memory buffer” is an organising heuristic for durability, not a claim that these systems share a single molecular mechanism. What they share is a functional property relevant to therapeutics: each can store a change induced around the time of delivery and influence the biological response long after the RNA itself is gone.

Table 1 makes the distinctions explicit. Adaptive immune memory resides in antigen-specific B and T lymphocytes and long-lived plasma cells, persists for months to years, and is measured by serology, germinal-centre activity and recall assays. Trained innate immunity resides in myeloid cells and their bone-marrow progenitors, is encoded epigenetically and metabolically over weeks to months, and is measured by chromatin accessibility, histone marks and cytokine recall (van Leent et al., 2021). Epigenetic state in non-immune cells operates on chromatin at disease-relevant loci. Regenerative repair operates at the tissue and niche level. Because these substrates differ, the framework does not assume that a carrier property which strengthens one will strengthen another; on the contrary, it predicts that memory capacity is substrate- and tissue-specific, a prediction we make explicit below.

**TABLE 1:** Biological memory systems relevant to RNA durability, with their distinct substrates, timescales and measurements.

Memory system	Cellular substrate	Timescale	Primary measurements
Adaptive immune memory	Memory B and T cells; long-lived plasma cells	Months to years	Serology; germinal-centre B cells; memory cell frequency; recall after challenge
Trained innate immunity	Myeloid cells; haematopoietic stem and progenitor cells	Weeks to months	Chromatin accessibility; histone marks; metabolic state; cytokine recall
Epigenetic state (non-immune)	Parenchymal and stem/progenitor cells	Weeks to indefinite	DNA methylation; histone modification; single-cell transcriptome
Regenerative repair	Tissue niches; resident progenitors	Variable; tissue-dependent	Tissue function; repair-cell phenotype; fibrosis; niche stability

The framework groups these systems by a shared functional role in durability, not by a shared molecular mechanism. Memory capacity is expected to be substrate- and tissue-specific rather than a single portable property.

### From RNA delivery to biological memory buffering

Delivery-centred RNA nanomedicine has achieved a great deal. Ionisable lipid nanoparticles protect mRNA, improve intracellular delivery and support cytoplasmic translation, while newer designs are beginning to address extrahepatic delivery and cell-specific targeting (Cheng et al., 2025; Hou et al., 2021; Lu et al., 2024; Saber et al., 2024). This work reinforces a useful point for the hypothesis: delivery platforms are not passive containers, because they reshape biodistribution, tissue exposure, pharmacokinetic behaviour and the immune context in which RNA is interpreted (Kulkarni et al., 2021; Nele et al., 2024). Still, the delivery-centred model tends to privilege early readouts. It asks where the RNA goes and how much protein is made. Those questions are necessary, but they are not enough for therapies whose value depends on persistence.

A memory-centred model asks a slightly different set of questions. Which immune or tissue states are created after RNA delivery? Does a formulation promote productive inflammation or mainly reactogenicity? Does antigen expression occur in a context that supports germinal-centre maturation, memory B cell persistence and T cell help? Does a gene-editing payload create durable correction without leaving behind chronic stress or immune clearance? In a regenerative setting, does transient RNA expression only give a short repair signal, or does it shift tissue behaviour into a more stable functional state?

The Biological Memory Buffer Hypothesis rests on the idea that therapeutic durability reflects a balance between biological forgetting and biological retention. Forgetting occurs when expression fades, antibody titres decline, edited cells disappear, reprogrammed cells fail to persist or a repaired tissue returns to a pathological baseline. Retention occurs when memory cells, corrected cell populations, stable expression programmes or repair states maintain the desired effect. Nanocarriers may influence this balance because they shape the context in which RNA instructions are delivered and interpreted.

### The biological memory buffer hypothesis

The Biological Memory Buffer Hypothesis states that programmable nanocarriers can improve the durability of mRNA vaccines and gene therapies by strengthening, stabilising or appropriately tuning biological systems that preserve, recall or maintain therapeutic information after RNA delivery.

First, durability is not determined by the RNA sequence alone. Modified nucleosides, untranslated regions, codon optimisation, poly(A) tail design and RNA purity influence translation and innate sensing, but long-term outcome also depends on the responding cell type, the tissue niche, the immune context and host biology (Pardi et al., 2018; Rohner et al., 2022). Two formulations carrying similar RNA cargo may therefore produce different durability profiles if they differ in biodistribution, inflammatory tone, cellular tropism or tissue retention.

Second, nanocarriers are biologically active interfaces. Lipid nanoparticles can stimulate innate immune pathways, interact with antigen-presenting cells and influence T follicular helper responses and humoral immunity (Alameh et al., 2021; Lee et al., 2023; Sharma et al., 2024). This can be useful for vaccines, where controlled immune stimulation may support memory formation. It may be less useful in repeated protein replacement or some genome-editing settings, where excess inflammation can reduce tolerability and complicate repeat dosing.

Third, biological memory can act as a therapeutic buffer. In vaccines, memory B cells, long-lived plasma cells and T cell memory can preserve protection even after circulating antibodies decline. In gene therapy, corrected cells, stable expression programmes and durable editing outcomes can buffer against disease recurrence. In tissue repair, stable cellular adaptation may buffer against relapse into injury or fibrosis. The therapeutic problem is therefore not only to create a response. It is to keep that response biologically available.

Fourth, the design target should shift from peak expression to persistence quality. Peak expression remains useful, especially for acute replacement or vaccine priming. But a high early signal that produces weak memory may be less valuable than a moderate signal that creates durable recall. This distinction matters for booster scheduling, chronic disease therapy, cancer immunotherapy and rare disease gene correction.

### Distinguishing a carrier-attributable memory effect

A central objection is that durability outcomes such as germinal-centre persistence and durable memory B and T cells reflect antigen biology and adaptive immunity, and so do not isolate a carrier-specific effect. We take this concern seriously, because without an isolation strategy the framework would risk relabelling known immunology. We therefore distinguish nanocarrier memory capacity from cargo design and from the established adjuvanticity of lipid nanoparticles by specifying both a conceptual definition and a control design.

Conceptually, nanocarrier memory capacity is the carrier-attributable component of durability: the difference in a durability endpoint between two formulations that are matched on cargo and on early exposure but differ in carrier properties. It is therefore defined as a contrast, not as an absolute property of a single formulation. If durability tracks only cargo, dose or antigen, the carrier-attributable component is zero, and that outcome would count against the hypothesis.

Operationally, the isolation requires a matched-comparison design. Two or more formulations deliver identical RNA cargo (the same nucleoside modification, untranslated regions, codon usage and poly(A) design) and are titrated to matched early exposure, defined as equivalent peak protein expression or peak antigen load and equivalent area under the expression curve over the first 24–72 h. Holding cargo and early exposure constant, the formulations are then allowed to differ only in carrier variables such as ionisable lipid identity, helper-lipid ratio, surface chemistry or size. Any divergence in downstream durability endpoints, measured longitudinally, is the carrier-attributable signal. Adjuvanticity is not assumed away; it is measured directly, through early innate activation and reactogenicity readouts, so that a memory difference can be attributed either to a difference in innate stimulation or to a distinct carrier effect. A formulation that raises durability solely by raising early innate activation would be informative but would not, on its own, establish a memory effect independent of adjuvanticity; the design separates these by matching, or explicitly co-varying, the innate-activation level.

This design also clarifies what would refute the hypothesis. If formulations matched on cargo and early exposure show no reproducible differences in persistence windows or memory biomarker profiles across adequately powered studies, then nanocarrier memory capacity, as defined here, does not exist as a separable determinant, and durability is better explained by cargo, dose, target-cell longevity and host context. The framework is constructed so that this is a realistic and reportable outcome, not a possibility excluded by definition.

### Why lipid nanoparticles are the right model system

Lipid nanoparticles are a strong model for this hypothesis because they are clinically validated and biologically active. Their components influence RNA encapsulation, organ tropism, endosomal escape, inflammatory signalling and manufacturing scalability (Cullis and Felgner, 2024; Hou et al., 2021; Nele et al., 2024). The same broad platform can be tuned for vaccination, transient protein expression, immune modulation or genome editing, which makes it useful for comparing early delivery with longer-term therapeutic memory.

The vaccine literature gives a concrete example. SARS-CoV-2 mRNA vaccines induced persistent germinal-centre responses in draining lymph nodes, with spike-binding germinal-centre B cells detected for at least 12 weeks after vaccination in a human study (Turner et al., 2021). Another study found durable memory B cell and T cell responses for at least 6 months after mRNA vaccination, even as antibody profiles continued to change (Goel et al., 2021). These findings show that mRNA lipid nanoparticle platforms can generate biological memory. They also show that durability is multidimensional. Antibody titres, memory cell persistence and variant protection do not always move together.

The same logic applies to gene therapy. CRISPR-based medicines have entered clinical practice, and exagamglogene autotemcel represents a major milestone for edited autologous haematopoietic cells in severe haemoglobinopathies (Laurent et al., 2024). More recently, an individualised *in vivo* base-editing therapy for a rare genetic disease demonstrated how quickly bespoke RNA-guided editing strategies can now be developed and delivered using lipid nanoparticles (Musunuru et al., 2025). This case also illustrates the payload-versus-outcome distinction: the delivery and editing activity are intentionally brief, while the intended benefit, a corrected genomic state, is meant to persist indefinitely. As RNA-guided editing moves further into *in vivo* settings, repeatable and tissue-directed delivery will become increasingly important. The harder question is not whether editing can happen once, but whether the corrected biological state persists safely and for long enough to matter.

### Therapeutic memory engineering

Therapeutic memory engineering means deliberately designing RNA delivery systems to promote long-term biological retention of therapeutic information. The concept moves beyond the simple aim of producing protein after mRNA delivery. It asks whether the delivery system can shape the conditions under which that protein signal is remembered.

In vaccines, therapeutic memory engineering may involve formulations that support antigen presentation, germinal-centre quality, T follicular helper activity and durable B cell maturation. In cancer immunotherapy, it may mean delivery systems that promote tumour-specific T cell memory rather than brief effector activation. In gene therapy, it may mean limiting inflammatory disruption while supporting stable correction of disease-relevant cells. In regenerative medicine, it may mean delivering RNA transiently but in a way that nudges tissue repair towards a more stable functional trajectory.

This is not speculation without a foundation. Lipid nanoparticles enhance humoral and T follicular helper responses in vaccine settings (Alameh et al., 2021). Nanomedicine has been proposed as a tool for regulating trained immunity because nanomaterials interact with myeloid cells and haematopoietic compartments (van Leent et al., 2021), and platform nanotechnologies have been engineered specifically to reach myeloid cells and haematopoietic stem and progenitor cells in the bone marrow, the very compartments in which innate memory is encoded (Hofstraat et al., 2025). The new step is to connect these observations to RNA therapeutic durability and to treat memory as an engineering endpoint, not merely as an immunological aftereffect.

### Trained immunity as a liability to be bounded, not only an asset

Because the framework invokes trained immunity as a possible durability asset, intellectual honesty requires treating it symmetrically as a risk. Trained innate immunity reprogrammes myeloid cells and their progenitors epigenetically and metabolically, and platforms that reach these compartments can imprint them (Hofstraat et al., 2025; van Leent et al., 2021). The same imprinting that might support durable host defence can, if poorly regulated, contribute to chronic or maladaptive inflammation, which is a genuine safety concern for repeat-dose lipid nanoparticle platforms.

The framework therefore treats innate memory as something to be bounded and monitored, not simply strengthened. Concretely, we propose that repeat-dose platform development include innate-memory safety readouts alongside durability readouts: longitudinal myeloid training signatures (chromatin accessibility and histone marks at inflammatory loci), cytokine-recall profiles after a standardised secondary stimulus, markers of progenitor-compartment skewing, and tolerability across repeated doses. A formulation that raises a memory durability metric while also raising a maladaptive-imprinting metric should be flagged, not rewarded. In this framing, a desirable nanocarrier memory profile is one that maximises the intended retention while keeping innate imprinting within a defined safety envelope.

### Nanocarrier memory capacity

Nanocarrier memory capacity describes the carrier-attributable ability of a delivery platform to support the formation, maintenance and recall of useful therapeutic memory, measured as defined above by matched comparison. It should be considered alongside traditional performance measures such as particle size, polydispersity, encapsulation efficiency, biodistribution and transfection efficiency.

To make the construct measurable rather than qualitative, we propose a normalised scoring approach. For a given indication and tissue, an investigator selects one primary durability endpoint (for example, persistence window in days, or the fraction of corrected long-lived cells at a fixed timepoint). For each formulation, the durability endpoint is expressed relative to a matched reference formulation with equivalent early exposure, yielding a memory-capacity ratio. A ratio above one indicates carrier-attributable durability gain; a ratio near one indicates none. Because durability endpoints differ in scale across indications, all comparisons are normalised to the matched reference rather than reported as raw absolute values, which allows cross-formulation comparison within an indication without falsely implying comparability across unlike indications.

A nanocarrier with high expression capacity but low memory capacity may produce impressive early protein levels and still deliver limited durability. A different formulation may produce lower peak expression yet generate stronger recall, broader B cell maturation or more stable cellular reprogramming. In a vaccine, memory capacity may be reflected by germinal-centre duration, memory B cell breadth, long-lived plasma cell output and tissue-resident T cell persistence. In gene therapy, it may be reflected by the proportion of corrected long-lived cells, stable target-gene expression, low immunogenicity and resistance to silencing or cell turnover.

Memory capacity is likely to be context-specific. A formulation that is desirable for vaccination because it activates innate immunity may be poorly suited for repeated protein replacement. A quieter formulation that reduces inflammation may improve tolerability but fail to provide enough immune instruction for vaccination. There is no single best memory profile. The useful profile is the one that matches the clinical task.

### The therapeutic persistence window

The therapeutic persistence window is the period during which a biological response remains strong enough to provide clinical benefit after RNA delivery. To function as a measurable endpoint rather than a label, it requires three specified elements: a measured durability quantity, a decay model fitted to that quantity over time, and an explicit clinical-benefit threshold below which the response is no longer considered protective or corrective.

For the decay model, we propose fitting longitudinal durability data to a small set of candidate functions and selecting among them by standard information criteria. A monophasic exponential decay describes a single dominant loss process; a biphasic decay describes a rapid early loss followed by a slower-decaying durable compartment, which is the expected shape when, for example, short-lived antibody-secreting cells decline quickly while long-lived plasma cells and memory cells persist. The persistence window is then the time at which the fitted curve crosses the prespecified clinical-benefit threshold. The threshold must be stated in advance and justified per indication, for example, a neutralising titre associated with protection for a vaccine, or a minimum fraction of corrected cells associated with phenotype rescue for a gene therapy. Reporting the decay slope and the half-life of each phase, rather than a single endpoint reading, allows formulations to be compared on the shape of their durability, not only on a single timepoint.

**FIGURE 1:**
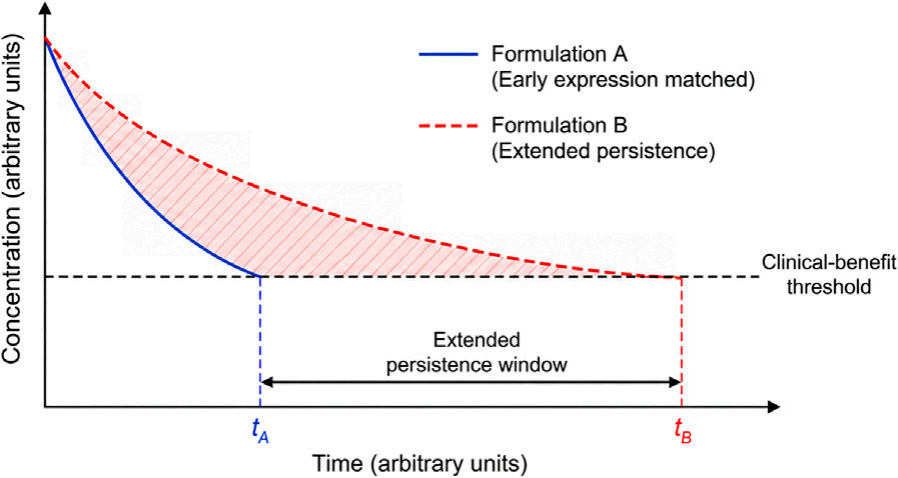
Illustrative, hypothetical decay curves for two formulations matched on early expression. Both reach the same peak, but Formulation B exhibits a larger slow-decay compartment, so its fitted curve crosses the clinical-benefit threshold later, giving a longer persistence window. The schematic is conceptual and contains no empirical data; it shows how matched-peak formulations can be distinguished by durability shape (Figure 1).

In vaccination, the persistence window is layered. Serum antibodies may decline first, memory B cells and T cells may persist longer, and protection against severe disease may outlast protection against infection (Arunachalam et al., 2023; Goel et al., 2021). As illustrated in Figure 1, two formulations matched for peak early expression may nevertheless exhibit distinct decay kinetics and therapeutic persistence windows. In gene therapy, the persistence window may depend on whether edited cells survive, expand or are replaced by uncorrected cells. Here the payload-versus-outcome distinction is decisive: the window is defined on the biological outcome, such as the durability of correction, not on the transient presence of the editing machinery. In regenerative medicine, the window may depend on whether transient RNA expression alters tissue-repair trajectories beyond the immediate expression period.

This concept can improve study design. Instead of asking only whether an RNA therapy works at day 7, day 28 or month 3, investigators should map when the effect begins to decay, which memory layer fails first and whether formulation changes extend the useful window. That would make durability a measurable design property rather than a vague claim.

### Is nanocarrier memory capacity portable across tissues?

A formulation optimised for the liver may not translate to spleen, marrow, muscle, lung, tumour or central nervous system, and memory biology itself differs markedly across these compartments. This raises a fair question: is nanocarrier memory capacity a portable property of a carrier, or must it be redefined per tissue? Our position is that it is not portable and should be defined per tissue and per indication. The matched-comparison definition is deliberately local; a memory-capacity ratio is meaningful only against a reference in the same compartment for the same endpoint.

This has a direct design consequence. Extrahepatic platforms that reach immune, bone-marrow, lung, spleen, muscle, tumour or central nervous system compartments should be evaluated for their memory consequences in those specific compartments, not assumed to carry over a hepatic result (Cheng et al., 2025; Hofstraat et al., 2025; Saber et al., 2024). The framework guides design precisely by forcing this question to be asked compartment by compartment, rather than by promising a single portable number.

### Carrier, host or interaction? Integrating host variability

Pharmacogenomic and host-context variability, including prior immune history, age, sex and genetic background, can materially shape durability. If so, the three constructs, which are formulation-centric as stated, might not be measurable as stable platform properties. We address this directly rather than leaving it to a caveat: durability is best modelled as a property of the carrier-by-host interaction, not of the carrier alone.

Practically, this means the constructs are estimated within host strata. The persistence window and memory-capacity ratio are reported for defined host contexts (for example, naive versus previously exposed, or stratified by relevant pharmacogenomic markers), and the carrier-attributable component is then the effect that remains after conditioning on host stratum. A platform property is stable only to the extent that the carrier ranking is preserved across strata; where rankings reorder across host contexts, the interaction term is itself the finding, and durability cannot be reported as a single carrier number. This makes host variability a measured dimension of the framework rather than an unmodelled threat to it.

## Functional memory endpoints as durability biomarkers

The Biological Memory Buffer framework requires endpoints that connect delivery biology with long-term outcome. Standard RNA nanomedicine endpoints, including protein expression, reporter signal, tissue biodistribution, cytokine induction and uptake, remain necessary. They should now be paired with memory-focused measures.

For vaccines, relevant endpoints include germinal-centre activity, memory B cell persistence, antibody breadth, T follicular helper responses, tissue-resident memory T cells and recall responses after antigenic challenge. For gene therapies, endpoints include stable editing frequency in long-lived cells, target-protein restoration, tissue-level functional correction, immune responses against delivery components and loss or persistence of edited cells over time. For regenerative applications, endpoints may include sustained tissue function, repair-cell phenotype, fibrosis recurrence, stem-cell niche behaviour and chromatin accessibility at repair-related loci.

Epigenetic and metabolic measures also matter. Trained immunity is regulated through epigenetic and metabolic reprogramming of innate immune cells and progenitor compartments (van Leent et al., 2021). If RNA nanomedicines influence these processes, then chromatin accessibility, histone marks, single-cell transcriptomes and metabolic state should form part of durability assessment, especially in immunotherapy and inflammatory disease research, and, as noted, also as safety readouts for maladaptive imprinting.

## A minimum durability reporting set

The most actionable contribution of this framework is a concrete, prioritised reporting standard. We propose that durability studies in RNA nanomedicine report, at minimum, a defined set of quantities so that formulations can be compared across laboratories and indications. Table 2 specifies this minimum durability reporting set. Adopting it would let the field move from comparing single early-expression readings towards comparing durability as a time-dependent, mechanism-linked property.

**TABLE 2:** Proposed minimum durability reporting set for RNA nanomedicine studies.

Reported quantity	Definition	Why it is required
Peak response	Maximum value of the primary durability quantity and its time of occurrence	Anchors early exposure and enables matched-peak comparison
Decay slope	Rate(s) of decline from the fitted decay model (monophasic or biphasic)	Captures durability shape, not a single timepoint
Persistence window	Time from delivery to crossing a prespecified clinical-benefit threshold	Converts durability into a clinically meaningful interval
Recall capacity	Response magnitude after a defined secondary challenge	Distinguishes retained memory from residual signal
Mechanism of loss or maintenance	Identified layer responsible (e.g., which memory compartment fails first)	Links durability to a biological cause and to design levers

Quantities should be reported per indication, per tissue and per host stratum. The set is intended as a floor for durability reporting, not a ceiling.

## The biological memory buffer framework across therapeutic domains

Table 3 maps the framework across therapeutic domains, identifying for each the relevant memory-disruption risk, functional memory endpoint, candidate buffer mechanism and translational significance.

**TABLE 3:** The Biological Memory buffer framework for RNA nanomedicine across therapeutic domains.

Therapeutic domain	Memory disruption risk	Functional memory endpoint	Potential buffer mechanism	Translational significance
Vaccine immunity	Rapid antibody decline or weak recall	Memory B cells, T follicular helper cells, tissue-resident memory T cells and recall responses	Nanocarrier-tuned antigen presentation and controlled innate activation	More durable protection and better booster timing
Gene expression	Short-lived protein expression or immune clearance	Duration of therapeutic protein expression and persistence of corrected cells	Lower inflammatory burden, improved cellular tropism and repeat-dose compatibility	Longer treatment intervals and improved chronic disease control
Genome editing	Transient editing activity without durable correction	Editing frequency in long-lived target cells and restoration of disease-relevant function	Targeted delivery to disease-maintaining cells with limited off-target inflammation	Stable correction with lower retreatment burden
Epigenetic and trained immunity	Uncontrolled inflammatory imprinting or weak innate memory	Chromatin accessibility, histone marks, myeloid training signatures and cytokine recall profiles	Programmable immunomodulatory delivery and dose-tuned innate sensing, with imprinting kept within a safety envelope	Balanced immune memory without chronic inflammatory toxicity
Regenerative repair	Temporary repair signals followed by relapse or fibrosis	Sustained tissue function, repair-cell phenotype and niche stability	Transient RNA expression paired with microenvironment-aware delivery	More durable tissue recovery after injury
Therapeutic persistence	Biological forgetting after the initial response	Persistence window, decay slope and functional recall after challenge	Memory-informed formulation, dosing and monitoring	Durability becomes a measurable design endpoint

The framework treats biological memory as a durability endpoint in RNA, nanomedicine. Biomarkers should be selected according to therapeutic class, disease context, route of administration and intended duration of effect, and reported per host stratum.

## Practical experimental implications

The hypothesis generates several testable implications. Comparative formulation studies should measure durability, not only early expression, and should adopt the matched-peak design described above so that any durability difference is carrier-attributable. Two lipid nanoparticles that show similar day-one transfection may diverge in memory-cell quality, inflammatory imprinting or persistence of therapeutic effect. Vaccine studies should integrate germinal-centre, memory B cell and T cell endpoints with conventional antibody titres. Antibody decline alone does not prove complete loss of protection, but it does show that researchers need to identify which layer of memory is carrying the response (Arunachalam et al., 2023; Turner et al., 2021).

RNA gene-therapy studies should also ask whether the target cells are short-lived or long-lived. Editing a rapidly replaced cell population may produce a narrow persistence window even when initial editing efficiency is high. Repeat-dose studies should become a core part of platform development because anti-particle immunity, inflammatory priming and tolerability can shape long-term feasibility, and because repeat dosing is exactly where maladaptive innate imprinting must be monitored (Lee et al., 2023; Sharma et al., 2024). High-resolution technologies should be used more routinely. Single-cell transcriptomics, spatial profiling, epigenomic assays and longitudinal immune phenotyping can show whether a delivery event creates stable memory or only transient activation.

Durability should be reported as a time-dependent function rather than a single endpoint, following the minimum durability reporting set in Table 2: peak response, decay slope, persistence window, recall capacity and the biological mechanism associated with loss or maintenance of effect. That level of reporting would help the field compare formulations across diseases instead of relying on early expression as a proxy for lasting benefit.

## Research priorities

Several priorities follow from the framework. The first is formulation memory mapping. Lipid nanoparticles and other nanocarriers should be compared not only by delivery efficiency but also, using matched-peak designs, by their carrier-attributable ability to generate durable biological states. The second is extrahepatic memory targeting. Because memory capacity is not portable across compartments, platforms that reach immune, bone-marrow, lung, spleen, muscle, tumour or central nervous system tissues should be evaluated for their memory consequences in those specific compartments as well as for targeting success (Cheng et al., 2025; Hofstraat et al., 2025; Saber et al., 2024).

The third priority is immune-context matching. Vaccines may benefit from controlled innate activation, while repeated protein replacement may require low immunogenicity. Cancer therapy may need stronger immune activation, whereas inherited-disease editing may require a calmer delivery environment, with innate imprinting kept within a defined safety envelope. The fourth priority is standardising durability metrics. The field needs shared language and shared thresholds for persistence windows, memory capacity, recall strength and retreatment thresholds; the minimum durability reporting set is offered as a starting point.

The fifth priority is clinical translation. RNA nanomedicine is moving quickly, and early clinical successes should not encourage premature generalisation. Durable memory in one vaccine platform does not guarantee durable expression in a rare-disease therapy, and a formulation that works in the liver may not translate to bone marrow, brain, lung or tumour microenvironments. Host variability also matters, because pharmacogenomic differences can alter immune response, metabolism, adverse-event risk and repeat-dose suitability; durability should therefore be reported as a carrier-by-host interaction. Submission-ready studies should avoid broad platform claims unless they include long-term, mechanism-linked durability data.

## Discussion

The Biological Memory Buffer Hypothesis reframes the purpose of RNA nanomedicine. Instead of viewing nanoparticles only as vehicles that move RNA into cells, it positions them as biological interfaces that can influence how therapeutic instructions are interpreted and remembered. This is a modest shift in language, but arguably a substantial shift in experimental logic. Many of the field’s biggest problems are not only delivery problems. They are persistence problems.

We are deliberately explicit about what is and is not new. The premise that delivery context shapes immune outcome is established (Alameh et al., 2021; Lee et al., 2023; Sharma et al., 2024; van Leent et al., 2021). The contribution here is to operationalise durability: to define carrier-attributable memory capacity as a measurable contrast, to specify a persistence window with decay models and prespecified thresholds, to propose a minimum durability reporting set, and to make the framework falsifiable through a matched-peak control design. The value is in measurement discipline and testability rather than in a newly discovered pathway, and readers should weigh it on those terms.

The framework also explains why the same technology can require different design logic in different clinical settings. In an mRNA vaccine, an immunostimulatory formulation may be valuable because it helps convert transient antigen expression into durable adaptive memory. In repeated mRNA protein replacement, the same immunostimulation may become a liability. In gene editing, transient expression is desirable for safety, but the corrected cellular state must persist, which is precisely why payload persistence and outcome persistence must be reported separately. In regenerative medicine, short-lived RNA expression may be enough only if it pushes tissue repair into a stable state.

There are risks in overextending the hypothesis. Biological memory is not always beneficial. Trained immunity may support host defence, but it may also contribute to inflammatory disease if poorly regulated, which is why this framework treats it as a liability to be bounded and monitored, with explicit safety readouts, rather than a quantity to be maximised. Epigenetic persistence can stabilise repair, but it can also stabilise maladaptive states. A mature research agenda must therefore treat memory as something to be tuned, measured and bounded rather than simply maximised.

The strongest value of the hypothesis is that it creates falsifiable predictions. If nanocarrier memory capacity is real, then formulations matched on cargo and early exposure should produce different persistence windows and memory-biomarker profiles. If therapeutic memory engineering is clinically useful, then memory-informed formulations should predict long-term outcomes better than peak expression alone. If the hypothesis fails, those matched comparisons will show no reproducible carrier-attributable differences, and durability will remain explained largely by cargo design, dose, target tissue and host context. Either outcome would clarify the field.

## Conclusion

RNA therapeutics have entered a new phase. The field has shown that mRNA, RNA vaccines and RNA-guided gene-editing payloads can be delivered effectively, but durable benefit remains uneven across indications and biological contexts. The Biological Memory Buffer Hypothesis proposes that programmable nanocarriers may shape the memory systems that determine whether therapeutic effects persist, decay or remain clinically retrievable after the initial delivery event.

In this model, biological memory is not decorative language. It is a measurable determinant of durability, defined as a carrier-attributable contrast, equipped with decay models, thresholds, a reporting standard and a control design that can refute it. Therapeutic memory engineering, nanocarrier memory capacity and the therapeutic persistence window offer a practical vocabulary for designing and testing next-generation RNA nanomedicines. The future of the field may depend not only on delivering better instructions, but also on helping cells and tissues remember the right instructions for long enough to matter.

**FIGURE 2:**
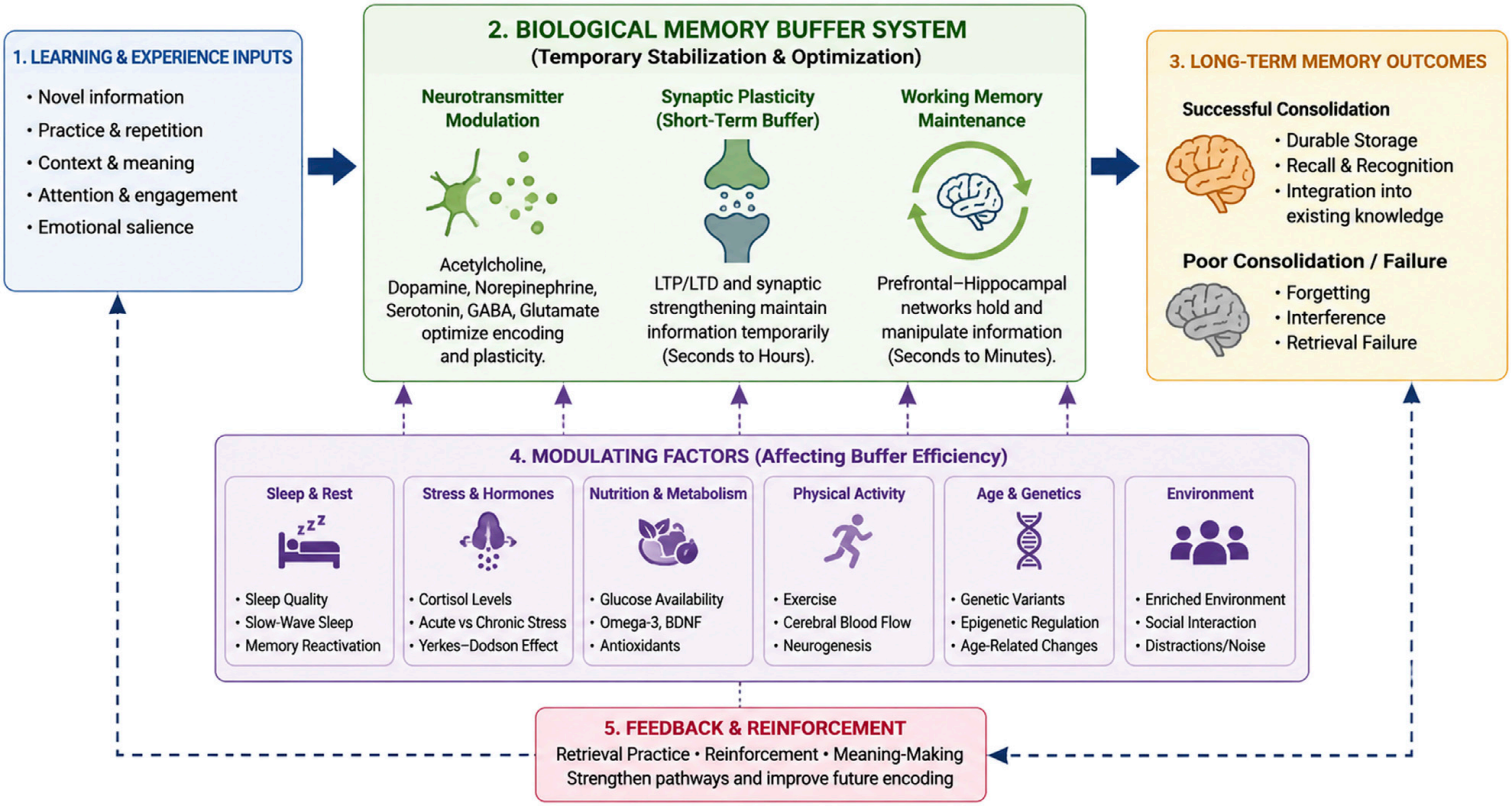
Conceptual framework of the Biological Memory Buffer Hypothesis.

The delivery event is shaped by nanoparticle composition, route of administration, biodistribution, cellular tropism, endosomal escape and innate immune activation. These factors interact with adaptive immune memory, trained innate immunity, epigenetic regulation, regenerative repair programmes and tissue-resident cellular states (Figure 2).

## Data Availability

The original contributions presented in the study are included in the article/supplementary material, further inquiries can be directed to the corresponding author.
